# The phorbol 12-myristate-13-acetate differentiation protocol is critical to the interaction of THP-1 macrophages with *Salmonella* Typhimurium

**DOI:** 10.1371/journal.pone.0193601

**Published:** 2018-03-14

**Authors:** Tregei Starr, Timothy J. Bauler, Preeti Malik-Kale, Olivia Steele-Mortimer

**Affiliations:** Laboratory of Bacteriology, Rocky Mountain Laboratories, National Institute of Allergy and Infectious Diseases, National Institutes of Health, Hamilton, Montana, United States of America; Duke University School of Medicine, UNITED STATES

## Abstract

THP-1 cells differentiated with phorbol 12-myristate 13-acetate (PMA) are widely used as a model for function and biology of human macrophages. However, the conditions used for differentiation, particularly the concentration of PMA and the duration of treatment, vary widely. Here we compare several differentiation conditions and compare the ability of THP-1 macrophages to interact with the facultative intracellular pathogen *Salmonella enterica* serovar Typhimurium. The results show that THP-1 macrophages differentiated in high concentrations of PMA rapidly died following infection whereas those differentiated in low concentrations of PMA survived and were able to control the intracellular bacteria similar to primary human macrophages.

## Introduction

In immunocompetent humans, *Salmonella enterica* serovar Typhimurim (*Salmonella* Typhimurium) causes self-limiting gastroenteritis. However, severe systemic disease can occur in immunocompromised patients, such as the elderly or very young, or those with advanced HIV/AIDS. What roles macrophages play in the human systemic disease is not well understood, but studies in murine and other animal models have shown that macrophages are crucial for extraintestinal survival and dissemination of *Salmonella* Typhimurim.

*Salmonella* Typhimurim has evolved multiple mechanisms to exploit innate immunity and establish systemic infection. For example, while cell death is used by the host to limit replication of intracellular bacteria, *Salmonella* can exploit this process to facilitate dissemination. Macrophages detect *Salmonella* by recognition of pathogen-associated molecular patterns (PAMPs) including lipopolysaccharide (LPS), flagellin, and components of the Type III secretion system 1 (T3SS1) [1]. Unlike LPS, which is an essential component of the outer membrane of *Salmonella* and is always present on the bacterial surface, expression of both flagellin and T3SS1 are tightly regulated both *in vitro* and *in vivo*. In vitro, macrophages infected with *Salmonella* induced for motility and/or invasion (T3SS1 and/or flagella are present) undergo pyroptosis, or inflammatory cell death, within 1 h of bacterial internalization [2,3]. By controlling the bacterial culture conditions, *Salmonella* Typhimurium populations that DO or DO NOT express these virulence factors can readily be obtained. How the infection proceeds thereafter is dependent on multiple factors, including: the transcriptional state of the bacteria; the activation state of the macrophages; and the mechanism of internalization [3–6].

Although the phenotypic diversity of macrophages cannot be reconstituted *in vitro*, cultured cells grown under well-defined conditions are important for studying the molecular mechanisms involved in host-pathogen interaction. The THP-1 human monocytic cell line has been widely used as a model to study human monocyte and macrophage biology and function [7–9]. The differentiation of THP-1 monocytes into macrophage-like cells can be induced by phorbol 12-myristate-13-acetate (PMA), 1 α, 25-dihydroxyvitamin D3 (vD3), or macrophage colony stimulating factor (M-CSF). PMA-stimulated differentiation is generally preferred for generation of cells with similarities to human peripheral blood mononuclear cell (PBMC) monocyte-derived macrophages [7]. Differentiated cells are adherent, have lower rates of proliferation, are more phagocytic, and generally have increased cell surface expression of CD11b and CD14 [9–11]. The conditions used to differentiate THP-1 cells are highly variable with PMA concentrations ranging from 10 to 400 ng/mL and the duration of treatment ranging from 1–5 days of continuous stimulation, which may be followed by at least 1 day of rest. [11–19]. Since small differences in culture conditions can affect the maturation of THP-1 cells [15,19], it is important to determine the most appropriate conditions for differentiation when using these cells as a model.

Studies using THP-1 cells as a model for human monocyte-derived macrophages (huMDM), have yielded conflicting data on the ability of *Salmonella* Typhimurium to survive and replicate in human macrophages, regardless of the mechanism of internalization [12,20]. The goal of this study was to re-assess the ability of *Salmonella* Typhimurium to survive and replicate in PMA-differentiated THP-1 macrophages. Using bacteria grown under conditions that do not induce the T3SS1, a working model for *Salmonella* infection of a human macrophage-like cell was developed.

## Results

### THP-1 morphology and surface receptor expression

**Fig 1:**
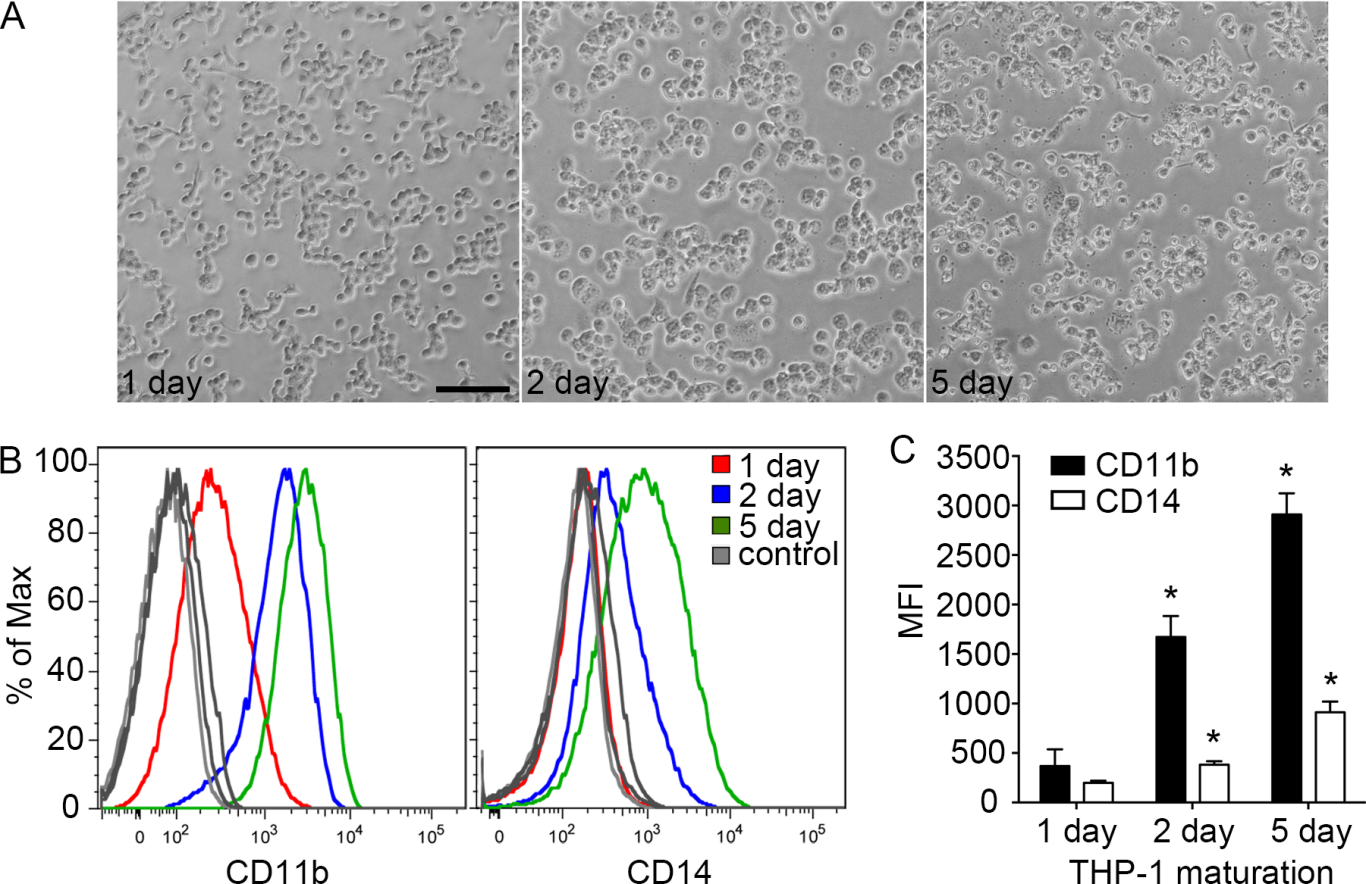
Surface receptor expression and cell morphology are dependent on differentiation conditions. (A) Representative bright field images of THP-1 cells differentiated for 1, 2, or 5 days in the presence of PMA (200 ng/mL). Scale bar is 100 μm. (B) Representative flow cytometric analysis of THP-1 cells stained using anti- CD11b or -CD14. (C) Quantification of mean fluorescence intensity (MFI) for CD11b (black) and CD14 (white). Data are means ± SD from three independent experiments. Statistical significance was determined using 1-way ANOVA with Dunnett post hoc test.

THP-1 cells were differentiated into macrophage-like cells (THP-1 macrophages) by incubation in the presence of PMA, which leads to a macrophage-like phenotype characterized by changes in morphology and increased cell surface expression of CD11 and CD14 [10,11,14,17,21]. Since both the PMA concentration and the duration of treatment affect maturation [19] we started by incubating THP-1 cells with PMA (200 ng/mL) either continuously for 1 or 2 days or for 2 days followed by 3 days of rest (5 day). This was based on a report that a minimum concentration of 100 ng/mL (162 nM) should be used for at least 48 hr [7]. As assessed by light microscopy, changes in cell morphology were dependent on the duration of incubation, with the cells becoming less refractive to light and larger over time (Fig 1A). Analysis by flow cytometry showed that surface levels of CD11b and CD14 were lowest in cells differentiated for 1 day and highest in cells differentiated for 5 days (Fig 1B and 1C). Since the 2- and 5-day PMA-differentiated cells appeared more macrophage-like than the 1-day cells, we used these conditions for the following experiments.

### *Salmonella* infection of differentiated THP-1 cells

**Fig 2:**
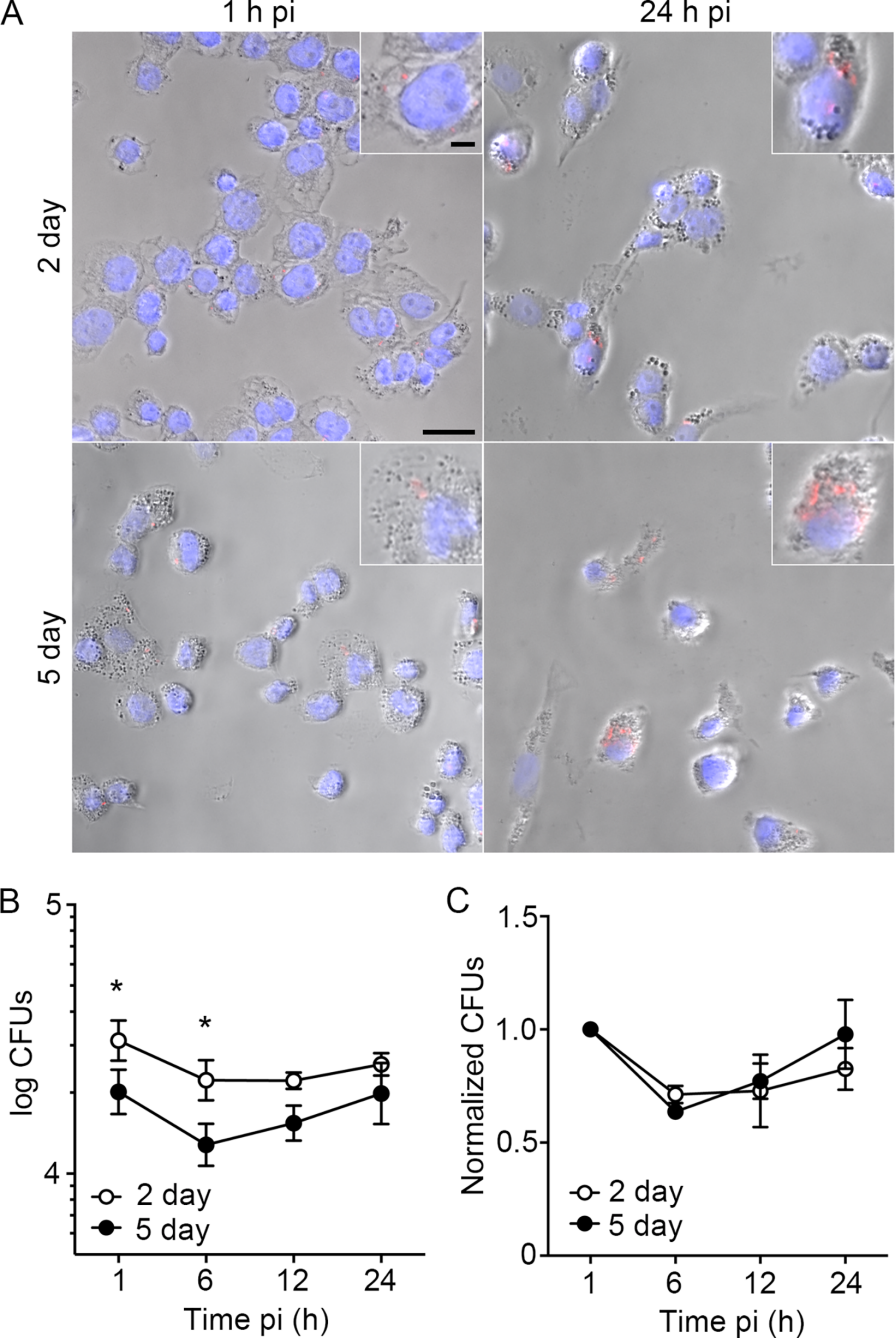
Replication of *Salmonella* in differentiated THP-1 cells. (A) THP-1 cells differentiated in PMA (200 ng/mL) for 2 days or 5 days and infected with mCherry-*Salmonella* (red) were stained with DAPI (blue) at 1 and 24 h pi. Scale bars are 25 and 5 μm (inset). (B, C) Intracellular growth of *Salmonella* in THP-1 cells differentiated 2 days (open circles) or 5 days (closed circles) at 1, 6, 12, and 24 h pi. (C) Growth normalized to 1 h pi. Data are means ± SD from three independent experiments. Statistical significance was determined using 2-way ANOVA with Sidak post hoc test.

We next assessed the ability of *Salmonella* Typhimurium (henceforth referred to as *Salmonella*) to survive and/or replicate within differentiated THP-1 macrophages. The bacteria were grown under conditions that suppress T3SS1 expression, thereby promoting internalization by phagocytosis (30 min) and suppressing T3SS1-induced inflammasome activation and cell death [5,22]. To facilitate single cell analysis, we used *Salmonella* constitutively expressing the red fluorescent protein mCherry (mCherry-*Salmonella*), so that live cell microscopy could be used to analyze bacterial numbers in infected cells. Differentiated THP-1 cells were infected at an MOI of ≈40:1 and the bacteria allowed to internalize for 30 min, after which extracellular bacteria were removed by washing. Under these conditions, approximately 10% of the cells were infected and whether the THP-1 cells were differentiated for 2 or 5 days, the majority of infected cells contained less than 5 bacteria at 1 h pi but by 24 h pi, cells containing greater than 5 bacteria were often observed (Fig 2A). For quantitative analysis of intracellular *Salmonella*, and to assess net intracellular replication, we used the same infection protocol but lysed cells at 1, 6, 12 and 24 h pi and quantified the viable intracellular bacteria by plating. Internalization appeared to be more efficient in cells differentiated for 2 days since, at 1 h, they contained ≈ 50% more bacteria than those differentiated for 5 days (Fig 2B). The numbers of intracellular bacteria then decreased so that ≈ 30% fewer bacteria were recovered at 6 h pi. Thereafter, there was either no net change in intracellular bacteria (2 day differentiated cells) or a slight increase (5 day differentiated cells) (Fig 2B and 2C). These results suggest that early (pre 6 h) loss of viable intracellular bacteria is followed by a period of either no, or minimal, net replication.

### THP-1 cell death during *Salmonella* infection

**Fig 3:**
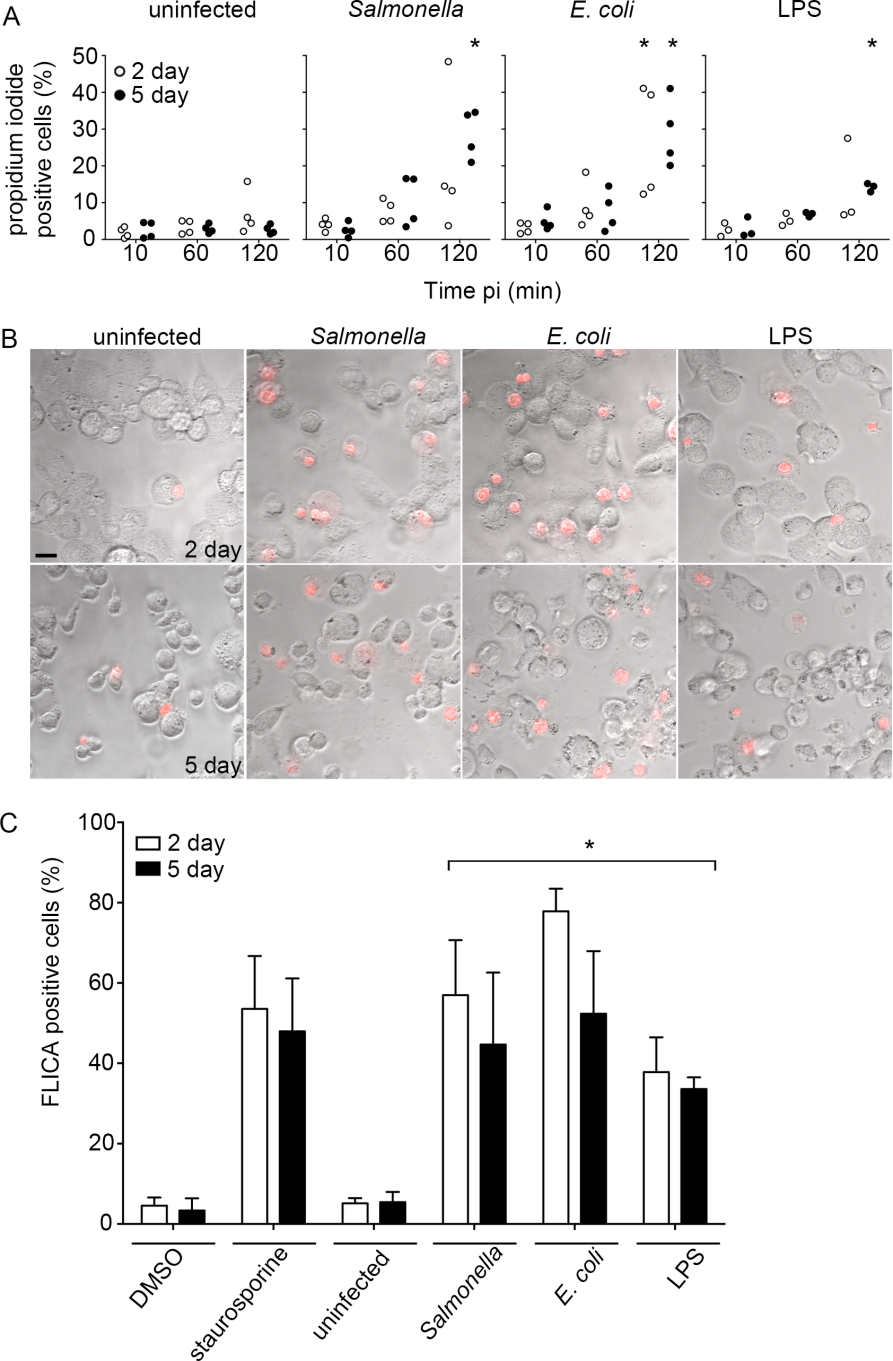
LPS induced cell death in differentiated THP-1 cells. (A) Quantification of percentage of propidium iodide positive THP-1 cells differentiated in the presence of PMA (200 ng/mL) for 2 days (white circles) or 5 days (black circles) following internalization of *Salmonella* or *E*. *coli* DH5α or a 30 min treatment with LPS (10 ng/mL). Data shown as individual experimental means. Statistical significance was determined as compared to uninfected controls using 2-way ANOVA with Tukey’s post hoc test. (B) Representative bright field images of THP-1 cells stained with propidium iodide (red) 120 min after internalization of *Salmonella* or *E*. *coli* or treatment with LPS. Scale bar 15 μm. (C) THP-1 cells were treated with DMSO or staurosporine (1 μM) for 3 h, infected with *Salmonella* or *E*.*Coli* for 30 min, or treated with LPS (10 ng/mL) for 30 min. At 2 h post-treatment cells were incubated with poly-caspase FAM-VAD-FMK before fixation, staining, and analysis. Data are means ± SD from three independent experiments. Statistical significance was determined using 1-way ANOVA with Dunnett post hoc test.

*Salmonella* can induce cell death in macrophages via several mechanisms. To assess THP-1 cell death, we used the membrane impermeant dye propidium iodide so that viable cells could be distinguished from dying cells by light microscopy. In mock infected cells, less than 10% of cells stained with propidium iodide. In THP-1 cells infected with *Salmonella*, cell death was markedly increased by 1 h pi and further increased by 2 h pi (Fig 3A and 3B). Interestingly, only ≈ 50% of the propidium iodide positive cells contained *Salmonella* at 2 h pi regardless of the duration of differentiation, suggesting the presence of a bystandard affect. Internalization of *E*. *coli* strain DH5α had a similar effect on levels of cell death indicating that *Salmonella* virulence factors are not required. Incubation of cells with purified *Salmonella* LPS (10 ng/mL), slightly increased the number of PI stained cells at 2 h vs 10 min or untreated control. Similar results were optained using a fluorescent pan caspase assay to assess caspase activity. In comparison to Staurosporine (1 μM) treatment for 3h, which resulted in ≈ 40–60% of cells staining positive, infection with either *Salmonella* or *E*. *coli* resulted in similar levels of induction (Fig 3C). LPS (10 ng/mL) treatment for 30 min stimulated caspase activation ≈ 40%. No significant differences were observed between the amount of cell death observed in 2-day and 5-day differentiated cells. Thus THP-1 cells differentiated with 200 ng/mL PMA undergo significant caspase associated cell death when either *Salmonella* or *E*. *coli* strain DH5α are internalized.

### Characterization of THP-1 cells differentiated in low concentrations of PMA

**Fig 4:**
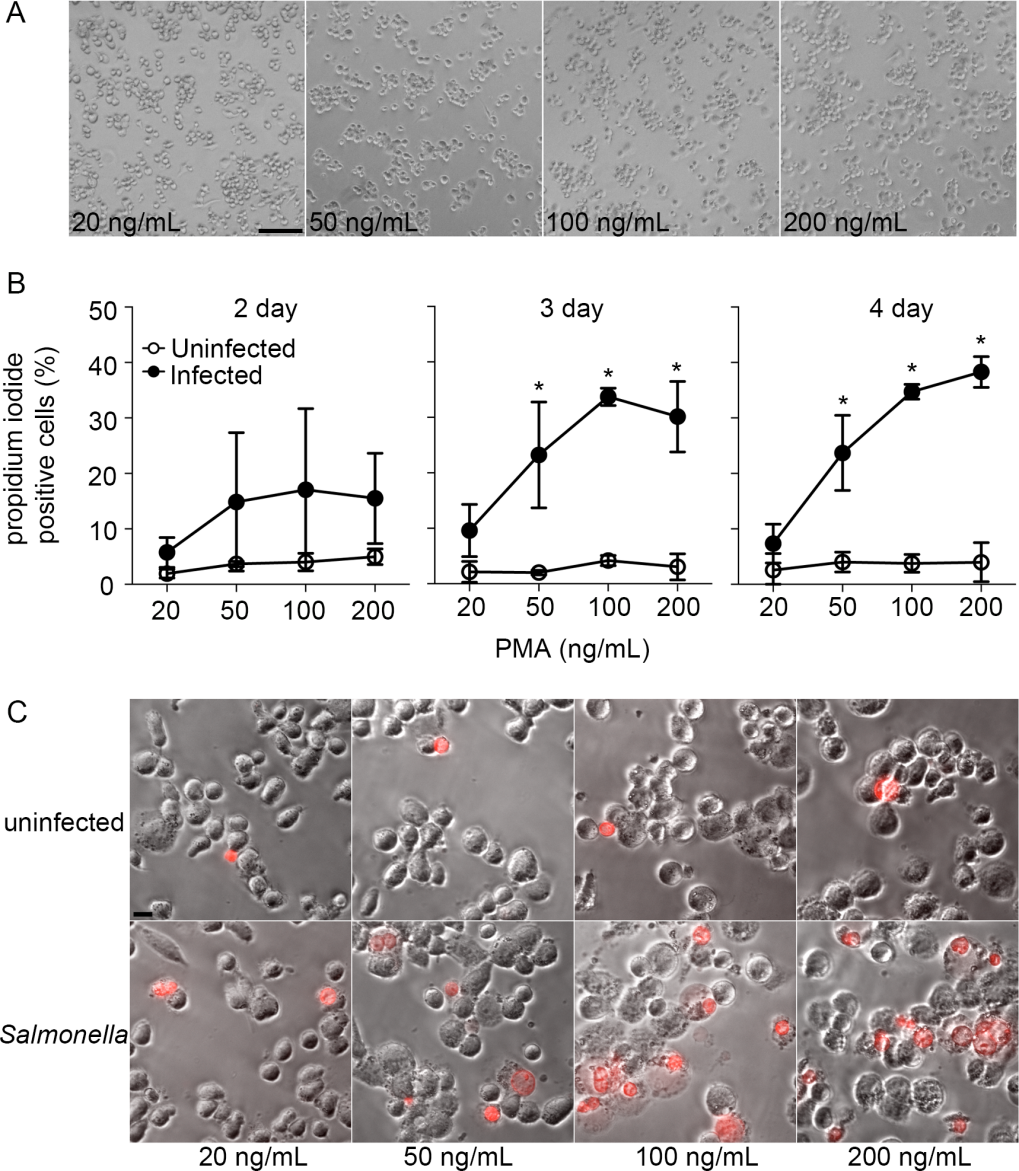
Cell death of *Salmonella*-infected THP1 cells is determined by PMA concentration. (A) Representative bright field images of THP-1 cells differentiated for 3 days in the presence of 20 ng/mL, 50 ng/mL, 100 ng/mL, or 200 ng/mL PMA, as described in Material and Methods. Scale bar is 100 μm. (B) Quantification of percentage of propidium iodide positive THP-1 cells differentiated for 2 days, 3 days, and 4 days at either 20 ng/mL, 50 ng/mL, 100 ng/mL, or 200 ng/mL PMA following infection with *Salmonella* (filled circle) for 2 h as compared to uninfected (open circles). Data are means ± SD from three independent experiments. Statistical significance was determined using 2-way ANOVA with Sidak post hoc test. (C) Representative images of differentiated THP-1 cells stained with propidium iodide (red) 2 h pi with *Salmonella* (bottom panel) or uninfected (top panel). Scale bar 25μm.

**Fig 5:**
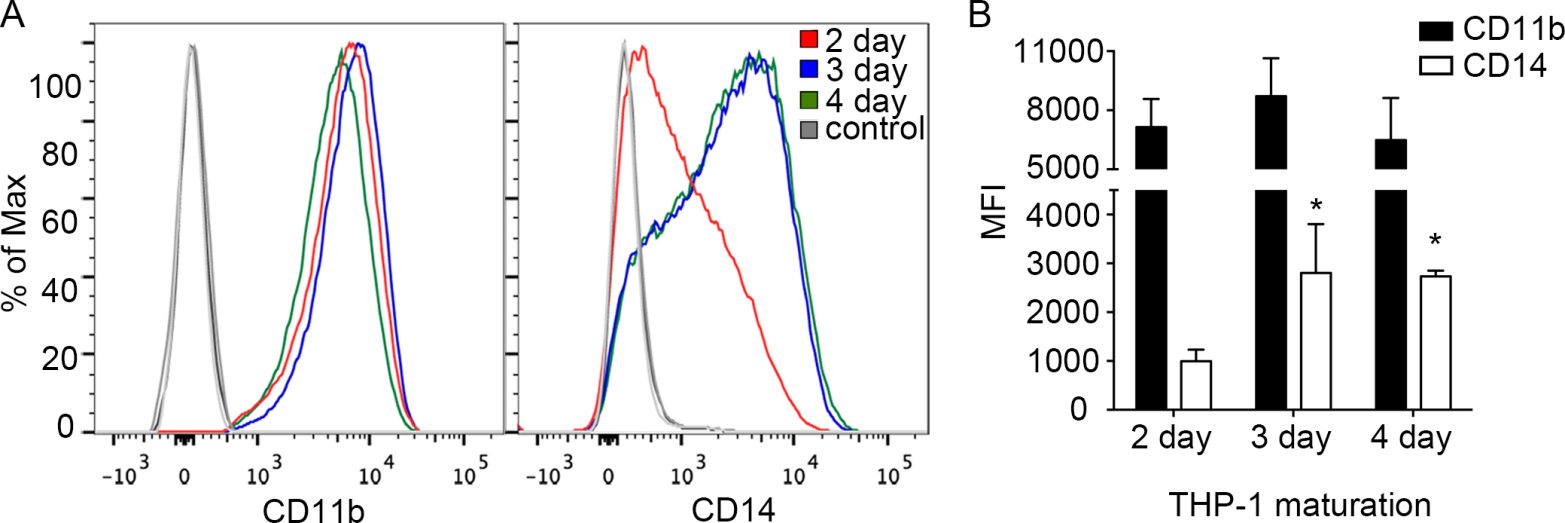
THP-1 surface receptor expression in cells differentiated with 20 ng/mL PMA. (A) Representative flow cytometric analysis of THP-1 cells stained using anti- CD11b or -CD14 antibodies. THP-1 cells were differentiated for 2 days (red), 3 days (blue), and 4 days (green) in the presence of PMA (20 ng/mL). (B) Quantification of mean fluorescence intensity (MFI) for CD11b (black) and CD14 (white). Data are means ± SD from three independent experiments. Statistical significance was determined using 1-way ANOVA with Dunnett post hoc test.

Although THP-1 cells are often differentiated in PMA at concentrations ≥100 ng/mL, lower concentrations may be sufficient to induce differentiation while minimizing off-target effects [14,15]. To see what role the concentration of PMA was playing in the cell death, we differentiated THP-1 cells in a range of concentrations of PMA (20–200 ng/mL) for 2, 3, or 4 days continuously, all of which induced adherence and spreading as indicators of differentiation (Fig 4A). Following infection with *Salmonella*, a clear dose-dependent effect on cell death was observed (Fig 4B and 4C). which was more pronounced in cells differentiated for 3 or 4 days. No significant cell death was detected in cells differentiated in the lowest concentration (20 ng/mL) of PMA. Flow cytometry analysis confirmed that cells differentiated in 20 ng/mL of PMA had increased levels of monocyte/macrophage markers CD11b and CD14 by 2 and 3 days of incubation, respectively (Fig 5). Together, these results suggest that THP-1 cells differentiated for 3 days in the presence of 20 ng/mL of PMA, exhibit classical surface markers of a macrophage and are less likely to undergo *Salmonella*-induced cell death compared to cells differentiated in higher concentrations of PMA.

### Comparison of *Salmonella* infection in THP-1 and monocyte- derived macrophages

**Fig 6:**
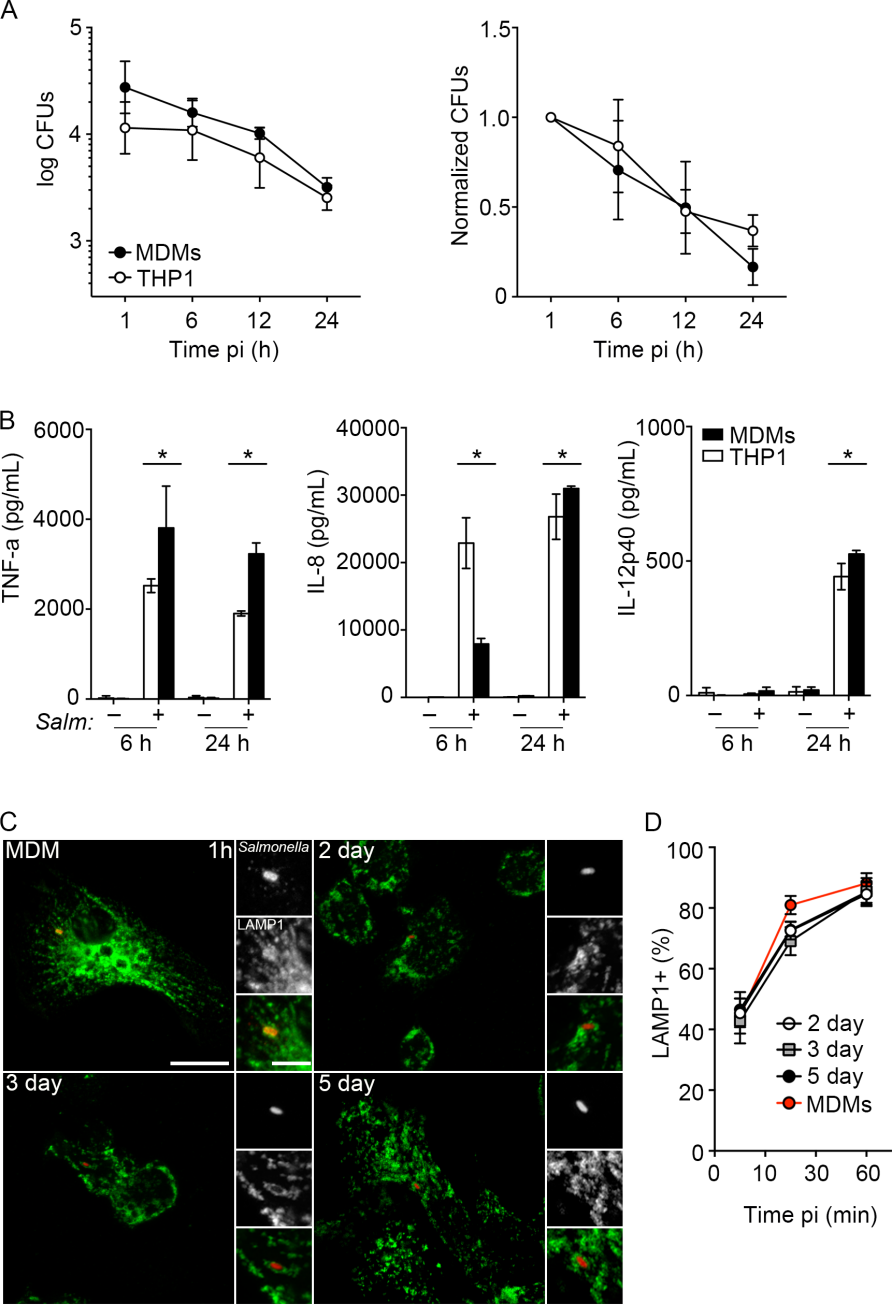
*Salmonella* are killed by THP1 cells and primary human macrophage cells. (A) Intracellular *Salmonella* in THP-1 cells differentiated for 3 days in the presence of 20 ng/mL PMA (open circles) or primary human monocyte derived macrophages (MDMs, closed circles) differentiated for 7 days in the presence of 100 ng/mL MCSF. Growth normalized to 1 h pi. Data are means ± SD from three independent experiments. (B) Cytokine expression as measured by ELISA for TNF-α, IL-8, and IL-12p40 in THP-1 cells (white bar) and human macrophages (black bars) in uninfected and *Salmonella*-infected cells at 6 and 24 h pi. Representative plots from three independent experiments. Statistical significance was determined as compared to the uninfected control for each strain using an unpaired *t*-test. (C) Representative maximum intensity projection images of *Salmonella* (red) infected MDMs and THP-1 cells differentiated in the presence of either 200 ng/mL PMA (2 day and 5 day) or 20 ng/mL PMA (3 day) at 1 h pi and stained for LAMP1 (green). Scale bars are 15μm and 5uM (insert). (D) Quantification of LAMP1 positive *Salmonella* in MDMs (red circles) and THP1 cells differentiated for 2 days (open circles), 3 days (grey squares), and 5 days (closed circles). Data are means ± SD from three independent experiments.

Our goal is to use PMA differentiated THP-1 cells as a model for human monocyte derived macrophages. Therefore, we next directly compared *Salmonella* infection in THP-1 cells, differentiated with 20 ng/mL PMA for 3 days, with primary human monocyte derived macrophages (MDMs) differentiated in the presence of macrophage colony-stimulating factor (M-CSF) for 7 days. These conditions have previously been used in our laboratory to generate non-activated human macrophages, which, when infected with *Salmonella* Typhimurium produce TNFα, IL-1B, IL-8 and IL-10 and support modest bacterial replication [6]. Using the gentamicin protection assay to assess the numbers of viable intracellular bacteria, we found that the numbers of intracellular bacteria decreased steadily between 1 h and 24 h pi in both THP-1 macrophages and human MDMs (Fig 6A). Non-activated human macrophages respond to infection with *Salmonella* by secreting cytokines such as TNF-α, IL-12, and chemokines including IL-8 (CXCL8) [6]. Here we found that these cytokines were secreted at similar levels from infected THP-1 cells and human MDMs. TNF-α and IL-8 secretion could be detected at 6 h pi and IL-12p40 was increased at 24 h pi. Intracellular trafficking of *Salmonella* through the endocytic pathway and acquisition of the late endosomal marker LAMP1 is another measure of *Salmonella* maturation during MDM infection. Comparison of LAMP1 acquisition kinetics for MDMs and THP1 cells, both those differentiated at 200 ng/mL and 20 ng/mL PMA, confirmed that vacuolar trafficking was similar between cell types.

## Discussion

THP-1 cells are widely used to study monocyte/macrophage function and biology. PMA is often used to stimulate differentiation into cells that mimic human MDMs, in terms of cell morphology, macrophage surface markers, and cytokine production [23]. However, the amount of, and duration of incubation with, PMA varies widely. Here we compared several different conditions for differentiation of THP-1 macrophages and how they affected the ability of the cells to interact with *Salmonella* Typhimurium.

Undesirable effects of high concentrations of PMA, have been reported and include activation towards the classically activated M1 state [15,24]. Here we found that differentiation of THP-1 cells in high concentrations of PMA ≥ 50 ng/mL) for 3–4 days results in high sensitivity to LPS resulting in significant cell death. In contrast, cells differentiated in 20 ng/mL of PMA were more resistant to rapid cell death when either exposed to LPS or infected with *Salmonella*. Furthermore, THP1 cells differentiated at the lower PMA concentration are more bactericidal, than those differentiated with 200 ng/mL PMA, and direct comparison of these cells with human MDMs, differentiated in the absence of activating signals, suggest that they have a similar ability to control intracellular *Salmonella*. The response of macrophages to *Salmonella* infection involves the secretion of cytokines such as TNF-α and IL-12 and chemokines including IL-8 (CXCL8) [6,25–27]. We found that THP-1 cells, differentiated in 20 ng/mL PMA for 3 days, and human MDMs released similar levels of these cytokines, although IL-8 may be released somewhat earlier by the THP-1 cells. Trafficking of *Salmonella* during MDM infection has been shown to involve the acquisition of the late endosomal/lysosomal membrane protein LAMP1 [28]. Our analysis also showed similar kinetics of LAMP1 acquisition in THP1 cells following *Salmonella* infection. Interstingly, this THP1 trafficking was similar regardless of the method used for differentiation, suggesting that *Salmonella* trafficking in these macrophage like cells is similar to the primary MDMs regardless of their differentiation state.

When *Salmonella* are internalized into macrophages there are a number of possible outcomes including, bacterial survival, replication, or persistence versus host cell death or survival. Our data show that differentiation in different concentrations of PMA can dramatically affect the host pathogen interaction. High concentrations of PMA likely induces some activation in THP-1 macrophages [15], which may account for the increased levels of cell death seen in these cells. Future experiments will address why *Salmonella* appear to replicate in a sub population of cells differentiated in high concentrations of PMA, and whether this is also true in cells differentiated in low concentrations of PMA. Similarly, further studies are required to confirm that the mechanism of killing of *Salmonella* in cells differentiated in low concentrations of PMA is similar to that occurring in the human MDMs.

*In vivo*, macrophages are plastic and heterogeneous cells. The local environment plays a large part in their differentiation and this can only be partially reconstituted *in vitro*. No cell line can accurately mimic these complex processes; nonetheless, judicious use of THP-1 macrophages can provide important mechanistic insights into the roles of human monocyte-derived macrophages. Our data, together with that of other groups, shows that the differentiation conditions must be carefully assessed within the context of the experimental question and that studies using other conditions need to be carefully considered when making comparisons.

## Materials and methods

### Ethics statement

Human peripheral blood monocytes, enriched by apheresis, were obtained from the NIH blood bank under protocol 99-CC-0168: “Collection and Distribution of Blood Components from Healthy Donors for In Vitro Research Use.” The protocol was reviewed and approved by the NIH Institutional Review Board (IRB). Signed informed consent was obtained from each donor, acknowledging that his or her donation would be used for research purposes by intramural investigators throughout the National Institutes of Health.

### Cell culture, bacterial strains, and reagents

THP-1 cells were obtained from ATCC (American Type Culture Collection: TIB-202) and grown in suspension in RPMI +Glutamax supplemented with 10% (v/v) FBS in a humidified 37°C, 5% CO_2_ incubator. All cell culture reagents were from Life Technologies, unless otherwise stated. Low passage (passage 15 or less) cells were plated in either 8-chamber Ibidi dishes (Ibidi) or 24-well tissue culture treated plates (Costar) in the presence of phorbol 12-myristiate-12 acetate (PMA, Sigma-Aldrich). For cells plated 5 days prior to infection, PMA was removed after 2 days of treatment. Primary human monocytes were isolated using negative selection (Dynabeads Untouched Human Monocyte Isolation Kit, Life Technologies) from apharesed whole blood from healthy donors and plated in 8-well Ibidi dishes 7 days prior to infection in RPMI supplemented with 5% (v/v) Male AB human serum (Sigma), 2 mM L-glutamine (Cellgro), 1 mM NaPyruvate (Cellgro), 1X MEM NEAA, 1 mM Hepes and 100 ng/mL macrophage colony-stimulating factor (MCSF) (Peprotech). Media was refreshed on day 3 and day 5. *Salmonella enterica* serovar Typhimurium SL1344 wild type [29] and constitutively expressing mCherry [30,31] have been previously described.

### Flow cytometry analysis of THP-1 differentiation

THP-1 cells were plated in 24-well tissue culture treated plates (Costar) as described above. Cells were removed from the well using TrypLE (Invitrogen) incubation for 10 min at 37°C. Cells were collected on ice and all subsequent steps were performed at 4°C. Cells were pelleted and resuspended in FACS buffer (0.1% azide, 2% fetal calf serum in PBS) containing 11 μg/mL IgG (Jackson). Cells were then stained with PerCP/Cy5.5 anti-mouse/human CD11b antibody (Biolegend, #101227) and eFluor 450 anti-human CD14 antibody (eBioscience, CD14 eFlour 450) before fixation in 2% (w/v) paraformaldehyde (PFA) and analyzed using flow cytometry. Analysis was done using FlowJo software (TreeStar, Inc. vX.0.7 and v9.5.2).

### Bacterial Infection, immunofluorescence and enumeration

Primary human macrophages and THP-1 cells were infected with *Salmonella* Typhimurium grown to stationary phase, 18 h at 37°C with shaking in Miller Lysogeny Broth (10 g/L Bacto tryptone, 10 g/L NaCl, 5 g/L Bacto yeast extract) (LB-M, US Biologicals) supplemented with antibiotic when necessary, as described previously [4]. Briefly, bacteria were pelleted at 8,000 × *g* for 2 min and resuspended in Hank’s Balanced Salt Solution (HBSS). A 1:20 dilution of bacteria in RPMI was immediately added to the cells at an MOI of 40. After 30 min at 37°C, cells were washed once with RPMI then incubated with media containing 50 μg/mL of gentamicin for 1 h, followed by incubation in 10 μg/mL gentamicin for the remainder of the experiment. For enumeration of bacteria, monolayers were solubilized in lysis buffer (0.2% deoxycholoate in PBS) and serially diluted in PBS before plating on LB-M plates. For immunofluorescence imaging, cells were fixed with 2.5% paraformaldehyde for 10 min before staining with LAMP1 (mouse monoclonal LAMP1-H4A3, DSHB) and Alexa Fluor 488 donkey-anti-mouse secondary (ThermoFisher).

### Microscopy

Brightfield microscopy was carried out on a Nikon Eclipse TS100 microscope with a 10X/0.25 NA Ph1 ADL objective and images were captured using a Nikon Sight DS-Fi1 camera. Fluorescent microscopy was carried out on a Nikon Eclipse TE2000 epifluorescence microscope using a Plan Fluor 40X/0.75 NA Ph2 DDL objective and the following emission and excitation filter combinations: DAPI (360/40_460/50), FITC (480/30_535/40), and TRITC (540/25_610/40) (Chroma Technology, Rockingham, VT). For propidium iodide the TRITC emission and excitation filter combination was used. A CoolSnap HQ2 14-bit digital camera (Photometrics, Tucson, AZ) was used to capture images. Confocal microscopy images were captured on a Carl Zeiss LSM 710 microscope using a Plan APOCHROMAT 63X/1.4NA objective. All post-acquisition image analysis was done using ImageJ software (W.S. Rasband, National Institutes of Health, Bethesda, MD) and Adobe Photoshop (CS5 v12.1 Adobe).

### Propidium iodide and FLICA assays

*Salmonella* or *E*. *coli* (MOI of 40), were cultured and internalized as detailed above. (*E*. *coli* culture conditions were identical to those used for *Salmonella* growth). Where indicated cells were treated with LPS from *Salmonella enterica* serotype Minnesota (10 ng/mL, Sigma) for 30 min. For propidium iodide assays, cells were incubated with propidium iodide (1 μg/mL, Life Technologies) for 15 minutes at the appropriate time post-infection and immediately analyzed by fluorescence microscopy without fixation. For each sample, several fields from duplicate wells were measured. For DAPI staining cells were fixed in 2.5% PFA for 10 min at 37°C before staining with DAPI (Life Technologies) for 10 min at RT and mounting in Mowiol (Aldrich).

For FLICA assays, control cells were treated with either vehicle (DMSO) or 1 μM staurosporine (Sigma) for 3 h prior to analysis. Cells were also incubated with *Salmonella*, *E*. *coli* or LPS. Cells were then incubated with poly-caspase inhibitor reagent FAM-VAD-FMK (Immunochemistry Technologies) for 1 h followed by a 10 min wash. Cells were immediately analyzed with several fields from duplicate wells being measured for all conditions.

### Cytokine assay

Supernatants were collected from uninfected and *Salmonella* infected primary human macrophages and THP-1 cells plated for 3 days in the presence of 20 ng/mL PMA at 6 h and 24 h pi. Cytokine secretion was measured by ELISA per the manufacturer instructions (R&D Systems).

### Statistics

All statistical analyses were done using Prism software (Graphpad Software v6.0g) and determined using ttest, 1-way, or 2-way ANOVA with appropirate multiple comparisons posthoc analysis. Stastical significance was determined to be p<0.05.
